# Open label safety and efficacy pilot to study mitigation of equine recurrent uveitis through topical suppressor of cytokine signaling-1 mimetic peptide

**DOI:** 10.1038/s41598-022-11338-x

**Published:** 2022-05-03

**Authors:** Caryn E. Plummer, Timothy Polk, Jatin Sharma, Sanghyo Sarah Bae, Olivia Barr, Amari Jones, Holly Kitchen, Michelle Wilhelmy, K. Devin, W. Clay Smith, Bryan D. Kolaczkowski, Joseph Larkin

**Affiliations:** 1 Department of Large and Small Animal Clinical Sciences, College of Veterinary Medicine, Institute of Food and Agricultural Science, University of Florida, Gainesville, FL 32611 USA; 2 Department of Microbiology and Cell Science, College of Agricultural and Life Sciences, University of Florida, Museum Road Building 981, PO Box 110700, Gainesville, USA; 3 Department of Ophthalmology Research, College of Medicine, University of Florida, Gainesville, FL 32611 USA

**Keywords:** Applied immunology, Autoimmunity, Cytokines, Immunological disorders, Immunotherapy, Inflammation, Signal transduction, Translational immunology, Eye diseases, Uveal diseases, Immunology

## Abstract

Equine recurrent uveitis (ERU) is a painful and debilitating autoimmune disease and represents the only spontaneous model of human recurrent uveitis (RU). Despite the efficacy of existing treatments, RU remains a leading cause of visual handicap in horses and humans. Cytokines, which utilize Janus kinase 2 (Jak2) for signaling, drive the inflammatory processes in ERU that promote blindness. Notably, suppressor of cytokine signaling 1 (SOCS1), which naturally limits the activation of Jak2 through binding interactions, is often deficient in autoimmune disease patients. *Significantly,* we previously showed that topical administration of a SOCS1 peptide mimic (SOCS1-KIR) mitigated induced rodent uveitis. In this pilot study, we test the potential to translate the therapeutic efficacy observed in experimental rodent uveitis to equine patient disease. Through bioinformatics and peptide binding assays we demonstrate putative binding of the SOCS1-KIR peptide to equine Jak2. We also show that topical, or intravitreal injection of SOCS1-KIR was well tolerated within the equine eye through physical and ophthalmic examinations. Finally, we show that topical SOCS1-KIR administration was associated with significant clinical ERU improvement. Together, these results provide a scientific rationale, and supporting experimental evidence for the therapeutic use of a SOCS1 mimetic peptide in RU.

## Introduction

Uveitis is a collection of sight-threatening intraocular diseases that are driven by aberrant inflammation from the immune system. Recurrent uveitis is characterized by repeated relapse (acute disease) and “remission” cycles of intraocular inflammation. In addition to relapse/remission cycles, some patients experience insidious disease where chronic subclinical disease drives ocular decrements^1^. Equine recurrent uveitis (ERU) is the leading cause of equine blindness, often resulting in euthanasia because of the dangers resulting from blind horses to themselves and their owners^2–4^. As such, a critical unmet need remains for ERU despite the efficacy of standard care, which includes the administration of topical steroidsClick or tap here to enter text. The reoccurring nature of the disease often results in patients becoming refractory to traditional therapy. Additionally, the prolonged use of steroids can produce many undesirable side effects, such as corneal disease (band keratopathy, delayed healing of corneal ulcerations and local immunosuppression) and predisposition to infection thus potentiating risks associated with daily use of steroids to alleviate inflammation^6^.

ERU is also the only known spontaneous model of human recurrent uveitis, which is a leading cause of ocular detriment within human patients. Uveitis accounts for 30,000 new cases of human blindness in the US per year and is responsible for 3–10% of all blindness worldwide^7^. ERU and recurrent human uveitis are autoimmune diseases driven by heavy influx of T lymphocytes into the uveal tract (which includes the iris, choroid, and stroma of the ciliary body) that produce high levels of destructive pro-inflammatory cytokines. Th1 (interferon gamma) and Th17 (interleukin (IL-6), IL23, IL17) phenotypes are present within the infiltrate and are thought to co-contribute to disease progression^8–12^. Consistent with the cellular infiltrates within the eye or ERU patients, a genome wide association study identified IL17A and IL17F as likely risk alleles for ERU^13^. Indeed, IL6-mediated necrosis of non-pigmented ciliary tissues is a common presentation in uveitis^14^. The influx of T cells (specific for ocular antigens) results in hypopyon (leukocytic exudate), photophobia, and edema^2^. As such, novel strategies to inhibit pro-inflammatory cytokine signaling, or T lymphocyte infiltration, would likely improve current standard of care for uveitis patients.

Although inflammation is essential for the elimination of pathogens and cancers, it must be regulated to prevent destruction of self-tissues. SOCS1, also known as STAT induced-STAT inhibitor 1, is an intracellular protein that regulates inflammation by limiting cellular responsiveness to cytokines^15^. SOCS1 operates in a classical feedback inhibition manner, in which SOCS1 protein is induced by a cytokine to limit the pro-inflammatory cascade mediated by that cytokine. SOCS1 is present in hematopoietic and non-hematopoietic cells and is highly conserved between vertebrate species, including humans, rodents, and horses^16^ SOCS1 is a potent regulator of several cytokines, including interferon gamma and IL6. SOCS1 possesses two well established mechanisms for the regulation of cytokine signaling: (1) a SOCS box, which targets intracellular proteins involved in the proinflammatory cascade to proteasomal degradation and (2) a kinase inhibitory region (KIR) that inhibits the function of kinases (jak2) involved in promoting the inflammatory signal. Mice deficient in SOCS1 (SOCS1-/-) die of a perinatal auto-inflammatory disease. In addition, SOCS1 deficiencies have been implicated within lupus, scleritis, multiple sclerosis, and asthma patients^17–20^ indicating that strategies that restore SOCS1 function may have efficacy in autoimmunity/autoinflammation^21^.

We have previously shown that peritoneal administration of a peptide mimic of SOCS1, SOCS1-KIR, inhibited progression of experimental autoimmune encephalomyelitis (a rodent model of multiple sclerosis), rescued SOCS1-/- mice from perinatal lethality, and reduced lupus pathologies in preclinical models^22–24^. In addition, we have shown that the topical application of SOCS1-KIR peptide in a simple eye drop form mitigated experimentally induced uveitis in mice and rats^5,25^. In this study using rodents, the topical administration of the peptide was demonstrated to be both safe and effective.

Current standard of care strategies for the treatment of recurrent uveitis (RU) are efficacious in most cases, however there is still an unmet need as unresponsive cases of RU are a leading cause of ocular morbidity in horses and humans. This pilot study tests the potential to translate previous studies, showing mitigation of induced experimental rodent uveitis, to spontaneous ERU clinical disease. In this study we demonstrate the high putative homology of the SOCS1-KIR mimetic peptide to the equine SOCS1 and putative binding of the SOCS-KIR mimetic peptide to the equine JAK2 protein. In addition, we show that topical administration of SOCS1-KIR was safe to the equine eye and was significantly correlated to reduced ERU-associated discomfort, hyperemia, and aqueous flare. Together these results of this pilot study justify more detailed analysis of the use of the SOCS1 mimetic as a novel therapeutic treatment for ERU.

## Materials and methods

### Conservation and phylogeny of SOCS1 KIR

The amino acid sequences for SOCS1 from 10 different vertebrate species were retrieved from National Center for Biotechnology Information’s (NCBI’s) protein database^26^ Of the 10 amino acid sequences, 6 were from mammals (*Homo sapiens, Rattus norvegicus, Mus musculus, Equus caballus, Bos taurus, Pan troglodytes*), 1 was from a bird (*Gallus gallus*), 1 was from a reptile (*Terrapene carolina*), 1 was from an amphibian (*Xenopus laevis*), and 1 was from a fish (*Danio rerio*). The 10 sequences were aligned using Rhône-Alpes Bioinformatics Center’s MULTALIN software^27^. Following the alignment, the sequences were exported to JalView^28,29^. In JalView, the aligned sequences were trimmed, leaving only the KIR domain. The KIR domain was then colored using the ClustalX coloring scheme. A logo of the trimmed KIR alignment was generated using the University of California Berkeley’s Web Logo tool^30^. The original SOCS1 protein sequences and the trimmed SOCS1 KIR sequences were imported into phylogeny.fr^31^. The default settings were used to generate two phylogenetic trees. The trees were exported in Newick format and annotated in FigTree v1.4.4^32^.

### In silico modelling of SOCS1 KIR and JAK2

Structural models of horse JAK2 (ENSECAP00000008048) bound to horse SOCS1 (ENSECAP00000022763) or the SOCS1 KIR peptide were constructed using MODELLER v9.23^33^. The structure of human JAK1 bound to human SOCS1 (6C7Y) was used as a template. Target sequences were aligned to template sequences using MAFFT v7.453 L-INS-i. 50 preliminary models of each complex were constructed and scored using the MODELLER objective function (molpdf), DOPE score and DOPEHR. Each score was scaled to units of standard-deviation across the preliminary models, and the 4 structural models of each complex having the best average of scaled scores were selected. The top-scoring models were used as starting points for replicate molecular dynamics simulations.

For each structural model, molecular dynamics simulations were executed using GROMACS v2019.2. The amber99sb-ildn force field and the tip3p water model were used. Initial dynamics topologies were generated using the GROMACS pdb2gmx algorithm with default parameters. Topologies were relaxed into simulated solvent at pH 7 using a 50,000-step steepest-descent energy minimization. The system was then brought to 300 K using a 50-ps dynamics simulation under positional restraints, followed by pressure stabilization for an additional 50 ps. Unconstrained molecular dynamics were run for 60 ns using a 0.002-ps integration time step, with the system sampled every 5 ps, following a 10-ns burn in. Simulations were run using Particle-Mesh Ewald electrostatics with cubic interpolation and grid spacing of 0.12 nm. Van der Waals forces were calculated using a force-switch cutoff of 0.8 nm. Nose–Hoover temperature coupling, with protein, and non-protein systems coupled separately and the period of temperature fluctuations set to 0.5 ps were also used. Pressure coupling was applied using the Parrinello-Rahman approach, with a fluctuation period of 1.0 ps. Non-bonded cutoffs were treated using buffered Verlet lists.

From each dynamics simulation, the central structure was inferred by calculating pairwise root mean square deviations between every pair of simulation samples and identifying the sampled structure most equidistant to the others, using the g cluster function in GROMACS. The root mean square fluctuation (RMSF) of each residue was measured over each dynamics simulation. Hydrogen bonds between SOCS1 or SOCS1 KIR and JAK2 were inferred using a radius cutoff of 0.35 nm and an angle cutoff of 30 degrees, and the proportion of simulation samples from which each residue formed a hydrogen bond with JAK2 was calculated. The minimum distance between each residue in SOCS1 or SOCS1 KIR and JAK2 at each sampled time point during the dynamics simulation was calculated, and the proportion of simulation samples from which each residue had at least 1 atom within 0.38 nm from an atom in the JAK2 protein was reported. Significance of differences in RMSF, hydrogen bonding and JAK2 contacts were assessed using the 2-tailed, 2-sample independent t-test, assuming unequal variances estimated across 4 replicate molecular dynamics simulations started from different structural models.

### Peptide synthesis

The SOCS1-KIR mimetic peptide (^53^DTHFRTFRSHSDYRRI) was purchased from GenScript®(Piscataway, NJ) at 95%purity. A palmitoyl-lysine (a lipophilic group) was added to the N-terminus of the peptides during the final step to assist in cell penetration. The peptide was characterized by high-performance liquid chromatography (HPLC) and mass spectrometry. The peptide was dissolved in Barnstead™ Nanopure™ water prior to use.

pJAK2 (^1001^LPQDKEYYKVKEP) was generated in-house using Applied Biosystems 431a automated peptide synthesizer (Applied Biosystems, Carlsbad, CA) by conventional fluorenylmethylcarbonyl chemical methods as described^34^.

### SOCS1-KIR binding assays

Direct binding assays were performed at room temperature (RT) for 2 h as previously described^34^ with modifications. Briefly, Polystyrene 96-well flat bottom plates were seeded with SOCS1 KIR peptide (500 µg/mL) in 0.05 M carbonate-bicarbonate buffer (pH 9.6). The plates were washed 4 times in the wash buffer (Phosphate-buffered saline in 0.05% Tween-20) followed by incubation for 2 h in blocking buffer (3% bovine serum albumin (BSA), 3 mM ethylenediaminetetraacetic acid (EDTA), 0.1% gelatin, and 0.05% Tween 20) and then washed 4 times. Serial dilutions of biotinylated phosphorylated JAK2 (pJAK2) in assay diluent (2% BSA, 3 mM EDTA, 0.01% gelatin, and 0.05% Tween 20) (10, 5, 2.5, 1.25, 0.625, 0.312 µg/mL) were then added to the coated plates for 90 min followed by washing 7 times. Streptavidin- horseradish peroxidase (1:4000, HRP) [BD Biosciences, Cat# 554066] was then added for 30 min followed by 12 wash cycles. Finally, 100 µL 3,3′,5,5′-Tetramethylbenzidine (TMB) substrate [BD Biosciences, Cat# 555214] was added for 30 min followed by assessment with spectrophotometer readings at an absorbance of 450 nM. For competitive Inhibition assays, 5 µg/mL biotinylated pJAK2 was pre-incubated with varying concentrations of SOCS1-KIR (500, 250, 125, 62, 31, 16, 8, 0 mg/mL) for 1 h at RT prior and then, a direct binding assay was performed.

### Evaluation of topical SOCS1 KIR for Safety in equine eye

To initially assess the safety of the peptide within the equine eye, four experimental, healthy horses (lacking ERU, 2 mares and 2 geldings) were purchased from a local horse dealer with a longstanding relationship with the University of Florida. The purchase was overseen by Animal Care Services and approved by the veterinarian in charge of the UF equine research program. The healthy horses were grade animals (mixed breed). A priori power analysis established that 4 equine subjects (eight total eyes) would be sufficient to assess a significant change in ERG measurements, based on historical data from the institution. No exclusion criteria were made in obtaining healthy, experimental horses. Investigator (C.P) was masked during administration, receiving deidentified peptide and placebo control groups. The horses initially received complete ophthalmic and physical examinations and received electroretinography (ERG) in both eyes to establish baseline profiles. Each of the four horses received 200 µg of SOCS1 KIR topically in one eye twice a day for two weeks or intravitreally once in one eye. Carrier control solution was administered in the other eye, serving as control. As such comparisons between eyes, either receiving peptide treatment or not, were made both within each individual horse and between horses to assess safety. ERGs, measuring a- and b-wave potentials and flicker responses were performed at days 0, 7 and 14. Complete physical and ophthalmic examinations were also performed at days 0, 7, and 14 to assess SOCS1 KIR mediated changes to ocular structure and function. At 14 days, after ERG and examinations, the experimental horses were euthanized humanely, by intravenous pentobarbital, for histopathological examination of the SOCS1 KIR treated eyes. In addition to isolating and submitting portions of the enucleated eyes to core facilities for independent evaluation, the ocular aqueous and vitreous components were collected and frozen for future use.

### Open label ERU study enrollment criteria

A priori ERU patients with confirmed ERU but lacking severe ophthalmic decrements (evidence of chronicity), were optimal for enrollment in clinical trial. However, given the unmet need presented by ERU, horses with uncontrolled ERU-mediated inflammation were also enrolled (even with ophthalmic decrement), given that peptide efficacy could be assessed. Reduction in hyperemia was established as the primary endpoint evaluation of peptide efficacy. A priori power analysis established that nine patients would be required to assess a significant decrease in hyperemia, based on historical data from the institution. Ten total patients were enrolled through direct contact with Dr. Plummer. Beyond No exclusions were made, with all participants noted in Table 1. All procedures were conducted on equine patients subsequent to owner consent.

**Table 1:** Horses used in study.

patient	Breed (sex Mare(M), Gelding (G),	Age (years)	Pathology/medical notes	Additional medication	Bilateral disease	Response to mimetic	Response to mimetic dryout period	Mimetic treatment duration
PT1	**Appaloosa (G)**	**21**	**Severe disease, blind OS, lens luxation, hyper-mature cataracts**	Diclofenac/atr opine	**yes**	** + + **	**yes**	**9 months**
Pt 2	Quarter hs (G)	16	Severe disease	Diclofenac/ atropine		–		4 months
Pt 3	**Miniature (G)**	**16**	**Severe disease**	**Monotherapy**	**yes**	** + + + **	**-**	**Lost to followup**
Pt 4	**Paint Mare**	**16**	**Moderate disease**	Atropine		** + + + **		2 months*
Pt 5	**Appaloosa (G)**	**17**	**Mild disease**	**Monotherapy**	**yes**	** + + + **	**yes**	8 months*
Pt 6	Appaloosa (G)	9	Severe disease	**Monotherapy**	yes	–		1 month
Pt 7	**Appaloosa (G)**	**16**	**Mild**	**Monotherapy**	**yes**	** + + + **	**yes**	3 months*
Pt 8	Quarter hs (G)	5	Severe disease	Diclofenac, atropine		–		2 months
Pt 9	**Appaloosa (M)**	**14**	**Moderate disease**	Diclofenac/atropine	**yes**	** + + + **	**yes**	8 months*
Pt 10	**Appaloosa (M)**	**18**		**Monotherapy**	**yes**	** + **		5 months*

### Evaluation of SOCS1-KIR efficacy in ERU horses

Ten horses (7 geldings and 3 mares) diagnosed with ERU were enrolled in a short-term, open-label trial to assess the efficacy of topical SOCS1 KIR. The administration of the SOCS1 KIR peptide was in the context of a therapeutic treatment. The horses of varied breed (appaloosa, quarter horse, miniature, and paint- aged 5–21) enrolled in the trial, presenting with acute or insidious disease, were admitted into the University of Florida Veterinary Hospital for 4–7 days to begin treatments with topical SOCS1 KIR. The horses admitted to the clinic were closely evaluated for efficacy and possible negative effects of the treatment. Examinations included intraocular pressure measurement, slit lamp biomicroscopy, and direct and indirect ophthalmoscopy as possible. 200 µg of SOCS1 KIR was administered in 100 µL topically, which was a small volume that prevented reflex tear formation and possible peptide elimination. Seven of 10 patients presented with bilateral disease for a total evaluation of 17 eyes to assess peptide efficacy (Table 1). After hospitalization, ERU patients were returned to their owners, who continued peptide administration from home. The horses underwent complete physical and ophthalmic examinations at day 0, day 3, day 14, and day 42. During the ophthalmic examinations, the horses were scored for discomfort, hyperemia, and aqueous flare using a modified MacDonald-Shadduck System^35^ by both the attending veterinary ophthalmologist and four additional masked veterinary ophthalmologists. Safety outcomes at each observation period included: (i) ocular irritation scores (utilizing the modified MacDonald-Shadduck scoring system-); (ii) number of new cases of ocular infections; (iii) number of horses affected with peptide-mediated damage to the eye; (iv) intraocular pressure (mmHg); and (v) ERG b-wave amplitudes to assess retinal function.

### IACUC statement

All procedures performed on animals were approved by the Institutional Animal Care and Use Committee (IACUC) of the University of Florida and were conducted in strict accordance of the approved guidelines.

This study did not adhere to the essential 10 ARRIVE guidelines as it was a pilot study to assess the feasibility of a larger, future study. Specific rationale is as follows: Given the novelty of the use of topical SOCS1-mimetic peptide, the administration of peptide in this pilot study was ethically conducted in open-label fashion, without randomization, so that rescue medications could be administered, if necessary. As, such blinding did not occur in the topical administration of the SOCS1-mimetic peptide to all 10 horses enrolled in the trial. A single investigator (C.P.) was aware of the drug allocations and the results of clinical evaluations. However, 4 masked veterinarians were utilized to obtain the clinical scoring obtained in Fig. 4E. Given the open enrollment utilized in this pilot study and the administration of drug by patient owners at home, confounders could not be controlled. Placebo medications were not utilized, although SOCS1 mimetic peptide was provided as compassionate use as specific owners noted symptom resumption during drug dry out period (not statistically analyzed).

### Statistics

Experimental data are presented as mean ± standard error of the mean (SD). All statistical analyses were performed using GraphPad Prism version 8 software (GraphPad Software, La Jolla, CA, USA). For the in vivo assessment of ERU recovery, paired students t tests were utilized. The data were deemed statistically significant if *P* ≤ 0.05. Linear, semi-logarithmic, and one site—specific binding curves were considered to have a good fit if R-squared ≥ 0.95.

## Results

### SOCS1 is highly conserved within vertebrates, and SOCS1-KIR peptide interacts with equine Jak2

**Figure 1:**
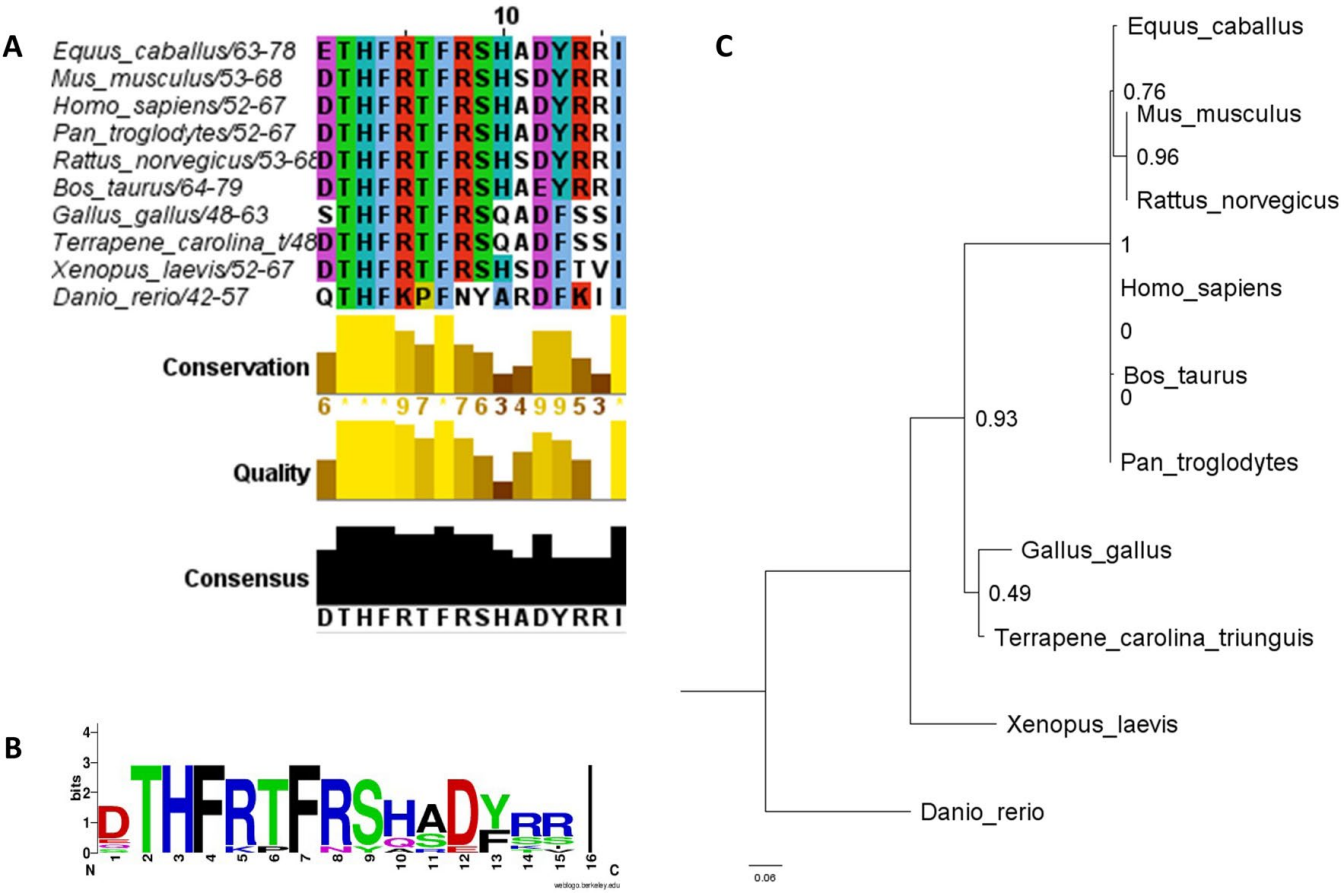
SOCS1 KIR Is Highly Conserved Among Vertebrates. (**A**) Multiple sequence alignment of 10 vertebrate SOCS1 sequences at the kinase inhibitory region (KIR) was retrieved from the National Center for Biotechnology Information’s (NCBI’s) protein database and aligned using PRABI’s MULTALIN tool. The residues were highlighted using the ClustalX coloring scheme. The amino acids contained within the KIR were evaluated according to conservation, quality, and consensus. (**B**) The sequences of the 10 vertebrate SOCS1 KIR domains, trimmed using Berkeley’s Web Logo tool, were used to generate a logo of the consensus sequence. (**C**) Full length SOCS1 from 10 different vertebrate species were used to generate a phylogenetic tree using Phylogeny.fr.

We have previously shown that the topical administration of a peptide corresponding to the kinase inhibitory region of murine SOCS1 (SOCS1-KIR), DTHFRTFRSHSDYRRI, was safe and prevented induced uveitis in murine and rat models of disease^5,25^. Given that horses and humans develop spontaneous recurrent uveitis, and that equine recurrent uveitis (ERU) is a major cause of sight deficits in horses, we proposed to assess the translatability of our rodent findings to equine disease. To predict efficacy of our SOCS1-KIR peptide in the treatment of ERU, we utilized bioinformatic tools to compare the similarity between our peptide mimetic and the SOCS1 kinase inhibitory region of several animal species. Ten SOCS1 protein sequences were retrieved from NCBI: 6 from mammals, 1 avian, 1 reptilian, 1 amphibian, and 1 sequence from bony fish. The sequences were used to generate a multiple sequence alignment using the MultAlin software. The multiple sequence alignment was then annotated in Jalview and the regions surrounding KIR were removed (Fig. 1A). This alignment demonstrated high amounts of conservation among all mammalian KIR sequences, with strong conservation among other non-mammalian vertebrates. Between the SOCS1 KIR mimetic peptide, and the KIR of *Equus caballus*, there were only two changes in amino acids. The first substitution was a conservative shift from glutamate to aspartate. The second difference was at the 11th amino acid position with a serine present in the mimetic peptide sequence compared to alanine in *Equus caballus.* This change to serine residue was found in several other species. Additionally, NCBI’s BLASTp^36^ analysis demonstrated 88% identity and 93% positive residues between the SOCS1-KIR mimetic and the corresponding sequence of SOCS1 from *Equus caballus*. The trimmed sequences were subsequently used to generate a logo of the consensus sequence (Fig. 1B). The sequence of the SOCS1 KIR mimetic peptide was identical to the consensus sequence except the serine to alanine shift at residue 11. Additionally, the 10 full length SOCS1 sequences were used to generate phylogenetic trees using Phylogeny.fr (Fig. 1C). The phylogenetic trees followed the generally accepted path of vertebral evolution, with closely clustered mammals forming a monophyletic group and *Danio rerio* (a fish species) being shown as the most distant related species. These results, in combination, demonstrate that the KIR region of SOCS1 is highly conserved among vertebrate species, particularly mammals, suggesting translatability of the SOCS1-KIR mimetic in the treatment of ERU.

**Figure 2:**
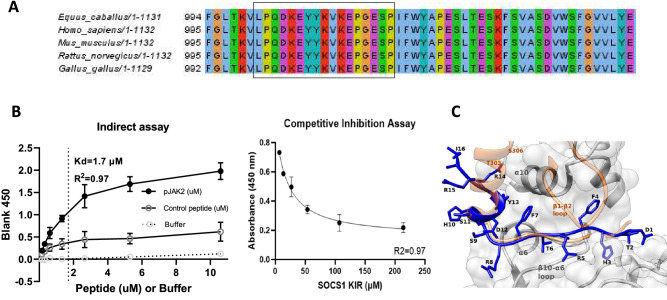
SOCS1-KIR Mimetic Peptide can Associate with Equine JAK2. (**A**) Graph showing direct binding of SOCS1 KIR mimetic peptide to biotinylated pJAK2 (1001–1013) peptide (n = 3). SOCS1 demonstrated a one site—specific binding relationship to pJAK2 (R^2^ = 0.97). (**B**) Representative curve of competition assay of biotinylated pJAK2 preincubated with varying amounts of SOCS1 KIR mimetic peptide for 2 h time, followed by addition to 96 well plate pre-coated with SOCS1 KIR mimetic (n = 3). This representative sample had a semi-logarithmic fit (R2 = 0.97). (**C**) Computer model generated using MODELLER overlaying the SOCS1 KIR mimetic (Blue) and full-length horse SOCS1 (peach) interacting with horse JAK2.

Endogenous SOCS1 binds to the activation loop of JAK2, inhibiting both autophosphorylation at tyrosine residue pY1007 and subsequent cellular programming that drives inflammation^37,38^. Given the homology of SOCS1-KIR mimetic to the corresponding region of equine SOCS1, we next assessed the conservation of Jak2 activation loop between putative horse, human, mouse, rat, and bird species. As can be seen in Fig. 2A the activation loop of Jak2, postulated to interact with SOCS1, is 100% conserved between the indicated species.

To confirm previous publications demonstrating the binding of the SOCS1-KIR mimetic peptide to the autophosphorylation site of pJak2 (Jak2 1001–1013)^23,34^, direct and competitive in vitro binding assays were conducted. The direct binding assay showed that biotinylated pJak2 (1001–1013), but not control peptide, bound to plate associated SOCS1-KIR mimetic peptide in a dose dependent manner which was denoted by increased absorbance, yielding a dissociation constant (Kd) of 1.7 μM (Fig. 2B). Competitive inhibition binding was next conducted, where a fixed concentration of biotinylated pJAK2 (1001–1013) sequence was preincubated with increasing concentrations of SOCS1-KIR mimetic peptide prior to incubation with plate bound SOCS1-KIR peptide. As can be seen in Fig. 2C, pre-incubation of pJak2 (1001–1013) with increasing concentrations of soluble SOCS1 KIR yielded a semi-logarithmic decrease in the signal intensity of pJak2 (1001–1013) binding to plate bound SOCS1-KIR peptide (R^2^ ≥ 0.95). These results were consistent with the previous studies showing SOCS1-KIR binding to the peptide pJak2 (1001–1013), which corresponds to the catalytic activation domain of the native peptide^39^, which is 100% conserved among humans, horses, rats, and mice.

We next utilized structural dynamics to evaluate the binding of SOCS1 KIR peptide to equine JAK2. The structural models of the SOCS1 KIR-JAK2 complex were inferred through homology modeling using the empirical structure of human SOCS1 bound to JAK1^40^. Four replicate molecular dynamics simulations of the SOCS1 KIR-JAK2 complex were conducted for 50 ns, with simulations of full-length equine SOCS1 bound to JAK2 used for comparison. SOCS1 KIR peptide was shown to bind to equine JAK2 in a conformation very similar to that of the N-terminal region of full-length horse SOCS1 (Fig. 2C and video). In the central complex obtained from molecular dynamics, the unstructured SOCS1 KIR N-terminal tail inserted into a largely hydrophobic cleft formed by the JAK2 β8-α6 loop as well as parts of α6 and α10 helices, with the short SOCS1 KIR α1-helix being stabilized by JAK2 α10^41,42^. This conformation was similar to what was observed in full-length SOCS1-JAK2 simulations and in the empirical human SOCS1-JAK1 complex. No strong structural clashes or repulsive interactions were observed between SOCS1 KIR and horse JAK2 in the native complex conformation. Together these results suggest that SOCS1 KIR likely binds horse JAK2 via a conserved, near-native structural interface.

### SOCS1-KIR peptide is safe for the equine eye

**Figure 3:**
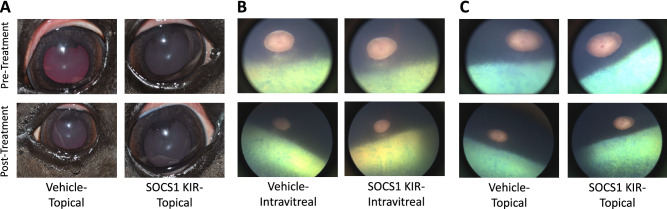
SOCS1 KIR Treatment Is Safe for the Equine Eye. Representative before and after photograph (**A**) and Fundus (**B**) images of equine eyes treated with either vehicle or topical SOCS1 KIR for 14 days.

To initially assess the safety of the peptide within the equine eye, two experimental, healthy horses (lacking ERU) were initially given complete ophthalmic and physical examinations followed by electroretinography (ERG) in both eyes to establish baseline ocular profiles. ERG is a well-established method of assessing gross physiological changes within an intact retina^25^. The horses then received 200 µg of SOCS1-KIR, or carrier topically in one eye twice a day for two weeks. ERG, measuring a- and b-wave potentials and flicker responses, and physical examinations were performed at days 0, 7, and 14 to assess SOCS1-KIR mediated changes to ocular structure and function. At 14 days, after ERG and examinations, the experimental horses were euthanized for histopathological examination of the SOCS1-KIR treated eyes. Significantly, physical examinations, ERG, and histological examination showed that SOCS1-KIR treated equine eyes were indistinct from the control eye or baseline readings (Fig. 3). As it was possible that topical SOCS1-KIR did not effectively penetrate the ocular barriers to affect the interior of the eye, the effect of intravitreal injection of SOCS1-KIR was evaluated in two additional horses. One single intravitreal injection of 200 mL (0.5 mg/ml) SOCS1-KIR was performed in one eye each of two horses. Again, horse evaluations at 7 and 14 days, followed by independent histopathologic evaluation of enucleated eyes, revealed no distinctions between SOCS1-KIR treated eyes versus control. Together, our results show that SOCS1 KIR administration was safe to the eye in either topical or intravitreal administrations.

### Topical SOCS1-KIR peptide mitigates equine recurrent uveitis (ERU)

**Figure 4:**
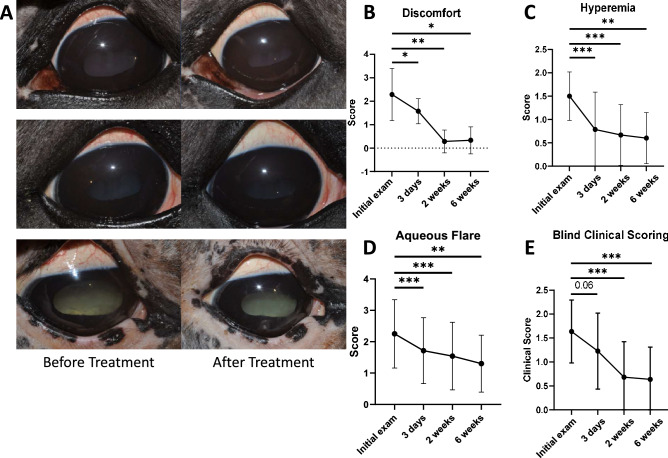
SOCS1 KIR Mitigates Equine Recurrent Uveitis Associated Pathology In Vivo*.* (**A**) Images of 3 representative horse eyes before and after treatment of SOCS1 KIR for 6 weeks (**B**) Average score of discomfort over the course of 6 weeks topical SOCS1 KIR treatment (n = 8). Horse eyes that did not exhibit any signs of discomfort were omitted from the analysis. (**C**) Average score of hyperemia over the course of 6 weeks topical SOCS1 KIR treatment (n = 14). (**D**) Average score of aqueous flare over the course of 6 weeks topical SOCS1 KIR treatment (n = 14). E) Images of 11 horse eyes at 4 different time points were provided to blinded veterinarians and clinically scored based on the criteria from the modified McDonald-Shadduck scoring system. (n = 8). *P*-values (∗ =  ≤ 0.05, ∗  ∗  =  ≤ 0.01, ∗  ∗  ∗  =  ≤ 0.001) were obtained by a paired two-tailed student’s t-test.

Given that topical and intravitreal SOCS1-KIR treatments were safe for experimental horses, we next conducted a pilot open-label trial where we tested our peptide treatment on equine patients possessing ERU. In our pilot trial 10 horses were enrolled, with a total of 17 eyes treated (Table 1). SOCS1-KIR was initially administered as a monotherapy to 5 patients with the remaining 5 receiving SOCS1-KIR in addition either atropine, or a combination of atropine and diclofenac (Table 1). The horses affected with acute ERU were hospitalized and treated with topical SOCS1-KIR (200 µg in 100 µl) twice a day at the University of Florida Veterinary Hospital for 4–7 days. During hospitalization, no horses experienced untoward treatment effects (i.e., ocular infections, irritation beyond what they experienced as part of their endogenous disease, corneal ulceration, ocular infections or declinations of comfort or sight.). After hospitalization, ERU patients were returned to owners who then continued peptide administration at home. Over a six-week period, most equine patients showed a visible reduction in hyperemia denoted in Fig. 4A, which reached a statistically significant 40% reduction in clinical scoring over the 6-week evaluation period (Fig. 4C *p* < 0.01), Additionally, a 50% reduction in aqueous flare (Fig. 4D) (*p* < 0.01), and a near complete reduction in overall discomfort (*p* < 0.05) (Fig. 4B) was observed over the same period. Given the open-label design of the clinical trial, we next conducted a masked veterinary scoring of randomly assorted ocular photographs from ERU patients taken either prior, or subsequent to SOCS mimetic peptide treatment. As can be seen in Fig. 4E, masked scoring yielded a statistically significant, 75% reduction in clinical scoring by 6 weeks of treatment (Fig. 4). Notably, one of the seven patients (patient 2) presenting with bilateral disease and minimal sight in one eye, had signs of very modest clinical improvement with SOCS1-KIR (2 mg/ml) administration with inflammation persisting. In this severe ERU patient, rescue medications in combination with SOCS1-KIR were unable to preserve sight, resulting in the euthanasia of the patient. However, resolution of inflammatory processes more strongly coincided with SOCS1-KIR administration in eight of the remaining 9 horses, although 4 horses did receive diclofenac and/or atropine which served to further alleviate pain associated with disease. Additionally, four horses received the topical (NSAID) diclofenac because of either persistent inflammation, or because they presented for evaluation already receiving medication (while still exhibiting clinical signs of inflammation that could be followed). It is significant, however, that all horses showed improvement without administration of the systemic NSAID flunixin meglumine or topical or systemic corticosteroids.

Due to perceived benefit of topical administration of the SOCS mimetic peptide, four owners requested continued use of the peptide at the conclusion of our initial study because of a noted return of inflammation and pathology during the peptide dry-out period. As such, peptide was made available to the owners as compassionate use. Although the results were not quantitated, as they were beyond the scope of our initial study, all owners receiving drug as compassionate care noted subsequent decreases in discomfort and inflammation when SOCS mimetic treatment was resumed. Moreover, no observed drug related negative events were recorded in the three horses receiving SOCS mimetic, administered twice a day for greater that 8 months (Table 1). Together, these data highlight the therapeutic potential of the SOCS1-KIR mimetic in ameliorating the signs, symptoms, and clinical features of ERU.

## Discussion

Equine recurrent uveitis (ERU) is a painful and debilitating autoimmune disease where activated T lymphocytes invade the normally immune privileged equine eye and promote the destruction of the uveal tract^1^. Despite the current standard of care treatments, which includes steroids, ERU remains a primary cause of ocular impairment in horses. Additionally, the prolonged use of steroids, as is often the case in the chronic disease ERU, often presents with additional negative side effects^43^. As such, an unmet need remains for better, safer, and more efficacious therapeutic strategies. Additionally, since ERU represents the only spontaneous model of human recurrent uveitis, therapies identified for ERU could likely be efficacious in human recurrent uveitis as well^1^. Although induced rodent models of uveitis do not mimic all the etiological processes that occur in spontaneous disease, they are immensely valuable in the elucidation of mechanisms of disease and tolerance, as well as the evaluation of novel therapeutic strategies^44^. Notably we have shown that a peptide mimic of the kinase inhibitory region of suppressor of cytokine signaling-1, administered as an eyedrop, was effective in both the induced anterior model of murine uveitis and the relapsing remitting rat model of pan-uveitis^5,25^. In order to test the possible translation of our rodent finding to the treatment of ERU in this pilot study we assessed the potential of SOCS1-KIR peptide to mimic endogenous equine SOCS1 interactions with Jak2, evaluated the safety of SOCS1-KIR topical administration to the equine eye, and tested the efficacy of SOCS1-KIR mimetic peptide treatment in a first-in-horse treatment of ERU. Although a pilot study, with limitations to be discussed in later paragraphs, in this manuscript we show striking results that strengthen a rationale for the use of topical SOCS1-KIR for the treatment of ERU and evidence of efficacy.

Acute episodes of ERU typically present with some or all of a host of clinical signs including ocular pain/discomfort, hyperemia, corneal changes, aqueous flare, hypopyon, and low intraocular pressure. Using a modified MacDonald-Shadduck System^35^ we observed statistically significant improvement in ocular discomfort, hyperemia, and aqueous flare in ERU patients receiving topical administration of SOCS1-KIR. Although not reaching statistical significance, we were encouraged by consistent improvements in hypopyon and intraocular pressure. The statistically significant decrease in the clinical score of aqueous flare relates to a decrease in inflammation and influx of inflammatory cells. Notably, our previous induced uveitis rodent studies demonstrated SOCS1-KIR mediated decreases in the chemokines CCL2, CCL20 and CXCL9, in addition to the chemokine receptors CCR2, CCR4, CCR6 and CXCR3^5,25^. Additionally, a recent study demonstrated significantly higher levels of the chemokine IP-10 (CXCL-10) in both the serum and aqueous humor of ERU patients compared to controls^45,46^. Future studies will assess chemokine regulation as a possible SOCS1-KIR mechanism of action. To properly validate the efficacy of SOCS1-KIR as a novel strategy for ERU, future studies will need to utilize a larger cohort of patients that includes a wider variety of horse breeds, as different breeds can experience distinct subsets of clinical disease. While Appaloosas often experience insidious disease characterized by persistent low-grade inflammation promoting cumulative disease, Warmbloods typically experience more acutely painful, classically waxing and waning manifestations as well as more posterior uveitis^47^. Of note, defective SOCS1 expression has been demonstrated in human scleritis patients^17^. It is therefore possible that endogenous SOCS1 expression and response may vary between different breeds of horses and ERU disease status. In our study we observed that the three patients (3/10) refractory to mimetic treatment had severe disease. This result may suggest a therapeutic window for mimetic peptide therapy. As such, a larger multi-center trial would assess equine breed specific effects of SOCS1-KIR treatment and help to establish a therapeutic window. A future clinical trial would also require control groups, which were missing from this pilot study. Although a placebo group was not included in this study, it is interesting to note that four equine patients had a return of clinical symptoms during the drug dry out period that were ameliorated by the resumption of treatment under compassionate use. Although not statistically significant, this result hints at very clear drug-specific effects. It was also striking that four of the ten patients in the trial showed responsiveness to SOCS-1 KIR administration as a monotherapy. Despite the limitations of this study, which were largely economic, we demonstrate that SOCS1-KIR administration was easily administered by owners as an eyedrop, safe to use, had no observed systemic immune suppression, and correlated with improved clinical outcomes in patients.

Recent studies have shown that T lymphocytes present in ERU patients have an activated phenotype, compared to control horses^48^. Many of the uveal tract destructive cytokines produced by infiltrating T lymphocytes are dependent on Jak2 for either production or signal transduction^38,46^. Through a combination of bioinformatics, in silico computer modeling, and direct peptide binding assays we show that the SOCS1-KIR mimetic peptide binds to equine Jak2 in a manner very similar to the kinase inhibitory region of endogenous SOCS1. Endogenous SOCS1 binds to the activation loop of JAK2, inhibiting the activation and nuclear translocation of STAT molecules thus limiting the magnitude and duration of the inflammatory signal^37,38^. Through bioinformatic analysis we also show that the activation loop of Jak2, postulated to interact with SOCS1, is 100% conserved between putative horse, human, mouse, rat, and bird species. In combination, these results allow us to extrapolate the likelihood that mechanistic insights revealed in rodent studies may indeed be translated to both equine and human disease. Using rodent models of uveitis, we have demonstrated that the topical administration of SOCS1-KIR to the eye suppresses both TH1- and TH17- associated cytokines, while promoting the production of IL-10^49^. It has been recently shown that elevated levels of IL-10 were present within the aqueous humor of ERU patients, compared to controls, and postulated to play a protective role^46^. As induced rodent models of uveitis have shown that either Th1 or Th17 T lymphocytes can promote experimental uveitis^10–12^, the ability of SOCS1-KIR to regulate both STAT1 and STAT3 signaling^38,50^ may be particularly valuable in inhibiting uveitogenic effector functions. Indeed, high levels of activated STATs were recently found within paraffin-embedded eye sections from ERU cases^51^. Additionally, we have previously shown that the peritoneal injection of SOCS1-KIR into a rodent model of SOCS1 genetic deficiency, or spontaneous lupus model, decreased circulating memory T cells and increased Foxp3 high T lymphocytes^22,24^. Together these results suggest that topical mimetic treatment may act through restoration of immunological tolerance within the eye. It is also possible that SOCS1-KIR acts through the inhibition of antigen presentation. Antigens are presented in the context of MHC-II to CD4 + T lymphocytes and facilitates inflammatory programming. Notably in ERU, MHC class II is upregulated within the nonpigmented ciliary epithelium, Müller cells, and retinal pigment epithelial cells^52^. Given that MHC class II expression is driven by inflammatory signaling promoted by cytokines such as interferon gamma, future studies using leukocytes isolated from the peripheral blood and ocular biopsies from ERU patients, combined with equine derived cell lines will be necessary to validate the mechanisms of action.

In summary, for the first time we present data providing a proof of principle for the safe use of a SOCS1 mimetic for the treatment of ERU and demonstrate a clear correlation between topical SOCS1-KIR administration to the equine eye of ERU patients and reduction of ERU associated symptoms within an open-labeled trial. As such, the data presented provide a strong impetus for detailed mechanistic evaluation of SOCS mimetic peptides as a novel treatment for recurrent uveitis.

## Supplementary Information

Supplementary Video 1.

## Abbreviations

ERU: Equine recurrent uveitis
SOCS: Suppressor of cytokine signaling
JAK: Janus kinase
KIR: Kinase inhibitory region
ERG: Electroretinography
IL: Interleukin
STAT: STAT1 signal transducer and activator of transcription
